# Occurrence and Exposure Assessment of Mycotoxins in Ready-to-Eat Tree Nut Products through Ultra-High Performance Liquid Chromatography Coupled with High Resolution Q-Orbitrap Mass Spectrometry

**DOI:** 10.3390/metabo10090344

**Published:** 2020-08-25

**Authors:** Alfonso Narváez, Yelko Rodríguez-Carrasco, Luigi Castaldo, Luana Izzo, Giulia Graziani, Alberto Ritieni

**Affiliations:** 1 Department of Pharmacy, Faculty of Pharmacy, University of Naples “Federico II”, Via Domenico Montesano 49, 80131 Naples, Italy; alfonso.narvaezsimon@unina.it (A.N.); luigi.castaldo2@unina.it (L.C.); luana.izzo@unina.it (L.I.); giulia.graziani@unina.it (G.G.); alberto.ritieni@unina.it (A.R.); 2 Laboratory of Food Chemistry and Toxicology, Faculty of Pharmacy, University of Valencia, Av. Vicent Andrés Estellés s/n, Burjassot, 46100 València, Spain

**Keywords:** almonds, pistachios, walnuts, mycotoxins, Q-Exactive Orbitrap, risk characterization

## Abstract

Tree nuts have become popular snacks due to their attributed benefits in the health state. Nevertheless, their susceptibility to fungal contamination lead to the occurrence of potentially dangerous mycotoxins. Hence, the aim of this work was to evaluate the presence of mycotoxins in ready-to-eat almonds, walnuts, and pistachios from Italian markets. The most relevant mycotoxin found in almonds was α-zearalanol in 18% of samples (*n* = 17) ranging from 3.70 to 4.54 µg/kg. Walnut samples showed frequent contamination with alternariol, present in 53% of samples (*n* = 22) at levels from 0.29 to 1.65 µg/kg. Pistachios (*n* = 15) were the most contaminated commodity, with β-zearalenol as the most prevalent toxin present in 59% of samples ranging from 0.96 to 8.60 µg/kg. In the worst-case scenario, the exposure to zearalenone-derived forms accounted for 15.6% of the tolerable daily intake, whereas it meant 12.4% and 21.2% of the threshold of toxicological concern for alternariol and alternariol monomethyl-ether, respectively. The results highlighted the extensive presence of *Alternaria* toxins and zearalenone-derived forms, scarcely studied in ready-to-eat tree nut products, highlighting the necessity to include these mycotoxins in analytical methods to perform more realistic risk assessments.

## 1. Introduction

Tree nuts have become a popular alternative to unhealthy snacks due to their attributed benefits. The intake of tree nuts has been related to a lower risk of suffering from cardiovascular diseases through several mechanisms, and they can also act as antioxidant suppliers [1,2,3,4]. According to the International Nut and Dried Fruit Council (INC), the annual production of tree nut products has increased over the last ten years, especially forn almonds, walnuts and pistachios, reaching a maximum of 4.6 million metric tons in 2019 and highlighting a global trend in tree nut consumption [5].

Nevertheless, tree nuts are susceptible to fungal growth that can occur for several reasons related to environmental factors, such as moisture and temperature. In addition, improper post-harvest practices and storage conditions can also promote fungal contamination [6]. As a consequence of these mentioned factors, mycotoxins could also be expected in crops. These are secondary metabolites produced by several filamentous fungi, mainly *Alternaria*, *Aspergillus*, *Claviceps*, *Fusarium*, and *Penicillium* spp., which can exert severe adverse effects including neurotoxicity, nephrotoxicity, immunosuppression, or carcinogenesis [7]. According to the carcinogenic potential, some mycotoxins have been included in the list of human carcinogens released by the International Agency for Research on Cancer (IARC).

In order to control the content of potentially dangerous mycotoxins in ready-to-eat tree nut products, the European Commission released the Regulation (EC) 1881/2006 [8] amended by Regulation (EU) 165/2010 [9] setting maximum limits (MLs) for certain mycotoxins. Almonds and pistachios cannot exceed 8 µg/kg for aflatoxin B1 and 10 µg/kg for the sum of aflatoxin B1, B2, G1, and G2, whereas MLs in walnuts were set at 2 and 4 µg/kg, respectively. However, different mycotoxins could be expected due to the susceptibility of tree nuts to fungal contamination. In this line, *Aspergillus* and *Fusarium* genera have been characterized as other major pathogens in tree nuts, so their secondary toxic metabolites could also be expected [6,10,11]. Alongside aflatoxins, relevant toxins from the *Fusarium* genus included in the IARC classification, such as T-2 or zearalenone, have been evaluated in tree nut products [12,13,14,15,16,17]. However, zearalenone-derived forms have been scarcely studied and, recently, the European Food Safety Authority (EFSA) highlighted the necessity to include these metabolites in risk assessment studies due to their estrogenic activity [18]. Furthermore, the presence of other mycotoxins-producing fungi such as *Alternaria*, able to produce the genotoxic compounds alternariol monomethyl-ether (AME) and alternariol (AOH), has been detected in tree nuts [11]. According to the EFSA CONTAM Panel, these mycotoxins have been scarcely studied, so the toxicological potential is still unknown, and seems to be major contributors to mycotoxin exposure in several commodities [19]. Therefore, there is a necessity to develop analytical methods able to detect and quantify these less studied mycotoxins, since they could also contribute towards overall exposure when consuming ready-to-eat nut products.

To overcome this, sensitive methods are required in order to detect low levels of mycotoxins occurring in tree nut products. In addition, environmental conditions, geographical area, or harvest practices can lead to a strong variety among products regarding mycotoxin occurrence, so constant monitoring based on sensitive and multi-analyte methods are required for having proper mycotoxin profiles. The most recent methods for detecting mycotoxins in tree nuts are based on liquid chromatography coupled with tandem mass spectrometry [12,13,14,15,16,17]. However, other alternatives are able to provide a more precise detection and quantification. The use of high-resolution mass spectrometry stands as the best alternative when performing simultaneous determinations of analytes. Identification based on full scan-all ion fragmentation analysis represents an improvement in mass accuracy when compared to traditional multiple reaction monitoring analysis based on triple quadrupole [20]. Furthermore, Q-Orbitrap offers a better performance than other high-resolution mass spectrometers for low *m*/*z* compounds, as most mycotoxins. Therefore, the aim of this work was to evaluate the presence of eighteen mycotoxins from different genera in ready-to-eat tree nut products (*n* = 54), including almonds, walnuts, and pistachios from Italian markets through ultra-high performance liquid chromatography coupled with high-resolution Q-Orbitrap mass spectrometry. To achieve this, a single QuEChERS-based extraction was validated in the three commodities. In addition, the risk characterization resulting from mycotoxin contamination in ready-to-eat tree nut products was performed in the Italian population for the first time.

## 2. Results

### 2.1. Analytical Method Validation

The proposed methodology was validated for the simultaneous detection and quantification of 18 mycotoxins in almonds, walnuts, and pistachios. Results are shown in Table 1 Linearity, expressed through correlation coefficient (R^2^), ranged from 0.9908 to 0.9998 for all compounds analyzed. The matrix effect was quantified through the percentage of signal suppression/enhancement (%SSE) ranging from 77 to 120%, from 74 to 148%, and from 82 to 149% in almond, walnut, and pistachio, respectively. There were only two analytes out of the range 80–120%: AME showed a %SEE of 148% and 149% in pistachio and almond, respectively, whereas %SEE for ENNB were 77% and 74% in almond and pistachio. In order to avoid miscalculation, matrix-matched calibration curves were used for quantification purposes. Despite the complexity of those matrices consisting mainly of proteins and fatty acids, a simple acetonitrile-based extraction including a clean-up step with C18 was enough to almost completely remove the matrix interference. The method also displayed a high sensitivity, with limits of quantification (LOQs) ranging from 0.20 to 0.78 µg/kg for all the analytes in each matrix. The recovery studies showed satisfactory results. For almonds, values ranged from 81 to 106% at the highest fortification level (20 µg/kg), from 73 to 95% at a medium level (5 µg/kg), and from 71 to 95% at the lowest level (1 µg/kg). Similarly, walnuts showed values from 79 to 105%, 70 to 99%, and 71 to 100% for the highest, medium, and lowest levels, respectively. Finally, recovery values for mycotoxins in pistachios ranged from 80 to 113%, from 70 to 105%, and 72 to 107% for the highest, medium, and lowest fortification levels. The precision evaluated through RSD_r_ and RSD_R_ was below 20% for all the analytes at three spiking levels.

Several multi-mycotoxin methods for tree nut products based on QuEChERS methodology have been recently published, as shown in Table 2. Although methodologies are focused on detecting aflatoxins since they are the only regulated mycotoxins in tree nuts, less attention has been put into *Fusarium* and *Alternaria* mycotoxins, which are a common genera causing fungal contamination in almonds, pistachios, and walnuts, as stated by Marín and Ramos [10] and Escrivá, Oueslati, Font, and Manyes [11]. In addition, ZEN-derived forms have also been here validated for their simultaneous detection, as recommended by the EFSA [18]. The main feature of the present methodology lies in its high sensitivity when compared to previous ones, with LOQs ≤ 0.78 µg/kg. Sensitivity also plays a key role when performing exposure assessment studies, allowing a more realistic analysis and avoiding underestimation of mycotoxins as highlighted by the EFSA.

### 2.2. Analysis of Real Samples

Up to nine different mycotoxins were detected and quantified in the here-analyzed samples. Results are shown in Table 3. At least one mycotoxin occurred in 33 out of 54 nut products. The most commonly found mycotoxins belong to *Fusarium* species, whereas *Alternaria* metabolites were also found in all the studied commodities.

Referring to almonds (*n* = 17), five mycotoxins were identified in 41% of samples. The most relevant compound was α-ZAL, a *Fusarium* toxin that results from the metabolism of its parental mycotoxin, ZEN. This toxin was present in 18% of the samples, ranging from 3.70 to 4.54 µg/kg. β-ZEL, another product from the metabolization of ZEN, was found in 12% of samples at low levels going from 0.46 up to 0.62 µg/kg. ZEN has also been studied in almonds, and contamination at 1.2 and 3.48 µg/kg was reported by Škrbić, et al. [21] in the two analyzed samples. According to the present results, ZEN metabolites resulted as the major contaminants in almonds and scarce literature regarding them is available. Since ZEN appears to be a common toxin in tree nuts, its metabolites could also be expected, as observed in this study. Despite not being a major fungus in almonds, *Alternaria* toxins have been usually reported. AOH was also quantified in 12% of samples ranging from 0.34 to 0.37 µg/kg. In a previous work conducted by Varga et al. [22], AOH was found in one sample (*n* = 8) at 1.5 µg/kg, more than three times the contamination here reported. Wang, Nie, Yan, Li, Cheng, and Chang [15] reported AOH contamination in four samples (*n* = 25) with a maximum level of 54.24 µg/kg. On the contrary, aflatoxins are one the most studied toxins in every nut typology. AFB1 was found in one sample at 0.45 µg/kg, complying with the regulation regarding maximum limits. Liao, et al. [23] reported contamination with AFB1 at 0.3 µg/kg in one almond sample (*n* = 9), similar to the concentration found in this study. Due to the toxicological potential of aflatoxins, special treatments are used in order to eliminate fungal contamination or to inactivate the toxins, such as roasting, sorting, and physical segregation [10], so it is not usual finding aflatoxins at concentrations above the maximum limits (8 for AFB1 and 10 µg/kg for the sum of aflatoxins).

Walnuts (*n* = 22) showed contamination with seven mycotoxins, finding at least one in 59% of samples. ZEN was quantified in 18% of samples ranging from <LOQ to 0.93 µg/kg. Several metabolites were also detected: β-ZEL was the most common one, present in 24% of samples varying from 0.3 to 0.55 µg/kg; β-ZAL was found in 18% of samples and, quantitatively, meant the most relevant one at levels going from 1.67 to 5.24 µg/kg; lastly, α-ZAL was present in 12% of samples at 2.13 and 2.24 µg/kg. ENNB1, an emerging *Fusarium* mycotoxin, was quantified at 1.3 µg/kg in one sample (6%). As previously observed in almonds, walnut samples also contained ZEN and ZEN metabolites. In the same study conducted by Wang, Nie, Yan, Li, Cheng, and Chang [15], a high contamination with ZEN was observed in one sample (*n* = 35) at 49.35 µg/kg, whereas Arroyo–Manzanares, Huertas–Pérez, Gámiz–Gracia, and García–Campaña [12] reported the presence of ZEN in one sample (*n* = 6) at 221.8 µg/kg. These levels of contamination strongly vary from those here obtained, with concentrations below 1 µg/kg. Furthermore, ZEN metabolites were quantitatively more relevant than ZEN, remarking the necessity of taken into consideration these mycotoxins when performing contaminant analysis in walnuts.

*Alternaria* toxins are not common mycotoxins included in tree nut studies. Nevertheless, AOH was extensively found at low concentrations ranging from 0.29 to 1.65 µg/kg in 53% of samples. AME was also present in 18% of samples at slightly higher levels, going from 1.13 up to 1.95 µg/kg. Therefore, sensitive analytical methods are required in order to understand the incidence of these toxins. In this line, Wang, Nie, Yan, Li, Cheng, and Chang [15] quantified AOH and AME in 23% and 31% of the walnut samples analyzed (*n* = 35), respectively, with a LOQ of 2 µg/kg. AOH was quantified in a range of 5.78–142.9 µg/kg and AME ranged from 1.53 to 110.5 µg/kg. Similar to the here-presented data, *Alternaria* toxins resulted as the most common toxins occurring in walnuts. 

Lastly, five mycotoxins were quantified in pistachios samples (*n* = 15), with at least one occurring in 80% of them. Among the matrices analyzed, pistachios resulted as the most contaminated. β-ZEL was the most prevalent mycotoxin, being detected in 59% of samples ranging from 0.96 to 8.6 µg/kg. α-ZEL and α-ZAL were both quantified in 12% of samples, ranging from 1.26 to 1.74 µg/kg and from 2.16 to 49.35 µg/kg, respectively. β-ZAL was only found in one sample at 11.86 µg/kg and, similarly, AOH was found at 7.75 µg/kg.

Quantitatively, pistachios showed a significantly heavier contamination (*p* < 0.05) when compared to almond and walnuts. In addition, the highest levels of AOH and ZEN metabolites were detected in pistachios. Although aflatoxins or ZEN have been analyzed in this matrix [22,24,25], there is scarce literature regarding the here-found toxins. Furthermore, the available validated procedures for ZEN metabolites cannot reach a high sensitivity as the here obtained (LOQ = 0.78 µg/kg), with Spanjer et al. [26] establishing the LOQs at 40 µg/kg and Hidalgo–Ruiz, Romero–González, Martínez Vidal, and Garrido–Frenich [17] at 1 µg/kg for α-ZEL. Sensitivity becomes a crucial feature in analytical procedures, even more when the contamination reported only reach a few µg/kg. In this line, Alcántara–Durán, Moreno–González, García–Reyes, and Molina–Díaz [16] did not find α-ZEL occurring in pistachios despite having a low LOQ (0.05 µg/kg). *Alternaria* toxins have not been deeply studied in pistachio neither. Varga, Glauner, Berthiller, Krska, Schuhmacher, and Sulyok [22] developed a procedure for AOH with the LOQ at 9.6 µg/kg, which would not have been sensitive enough for detecting the contamination here reported (7.75 µg/kg).

Co-occurrence of mycotoxins was observed in the three different commodities, as shown in Table 4. Walnuts presented a wide variety of combinations, including AOH in most of them. In total, co-occurrence happened in a 45% of walnuts samples. On the contrary, pistachios and almonds showed a lower incidence of co-occurrence, accounting for 27% and 1% of the total samples, respectively.

There is a strong variability referring to the content of mycotoxins depending on several parameters including temperature, moisture, or pre- and post-harvest practices, among others. This variability observed highlights the necessity of constantly monitoring these kinds of products using highly sensitive analytical procedures in order to ensure a safe consumption. Furthermore, an investigation carried out by the EFSA [18] considered the application of potency factors for ZEN metabolites ranging from 0.2 to 60 times the toxicity associated with ZEN. Considering the high uncertainty related to these metabolites, the analytical procedures should include these mycotoxins that could account for future exposure assessment studies.

### 2.3. Exposure Assessment

An exposure assessment and risk characterization were performed taking into consideration the left-censored data for the more prevalent mycotoxins detected in the samples, including ZEN-derived forms, AOH and AME. Results are showed in Table 5.

The tree nut consumption did not vary much throughout the age groups, so children with the lowest body weight showed the heaviest exposure. The percentages of relevant TDI or TTC calculated for children were two-fold higher than those for teenagers and adults, but below the maximum tolerable values established by the Scientific Committee on Food of the European Commission at 0.25 µg/kg bw/day for the sum of ZEN and its metabolites and 0.0025 µg/kg bw/day for both AOH and AME.

Under the worst-case scenario, the percentages of relevant TDI or TTC calculated for children meant between a tenth and a fifth of the established safety levels. Nevertheless, it might be a concern since ZEN, its derived forms, AOH and AME, can be found in different commodities such as cereals and other vegetal products, which are more commonly consumed by children.

The results evidence a negligible exposure of these mycotoxins due to ready-to-eat nut products, but these scarcely studied mycotoxins might be of importance when performing risk assessment through total diet studies.

## 3. Conclusions

An analytical method based on QuEChERS extraction and ultra-high performance liquid chromatography coupled with high-resolution mass spectrometry was validated for the simultaneous detection of eighteen mycotoxins in ready-to-eat almonds, walnuts, and pistachios. The features fulfilled the requirements set by the European Union regarding selectivity, linearity, trueness, precision, with high sensitivity based on limits of quantification. The procedure was then applied to almonds (*n* = 17), walnuts (*n* = 22), and pistachios (*n* = 15) acquired from Italian markets. The most relevant mycotoxin in almonds was α-zearalanol, found in 18% of samples (*n* = 17) ranging from 3.70 to 4.54 µg/kg. Walnut samples showed frequent contamination with alternariol, present in 53% of samples (*n* = 22) at levels from 0.29 up to 1.65 µg/kg. Pistachios were the most contaminated commodity, with β-zearalenol as the most prevalent toxin present in 59% of samples ranging from 0.96 to 8.60 µg/kg. In the worst-case scenario, the exposure to zearalenone-derived forms accounted for 15.6% of the tolerable daily intake, whereas it meant 12.4% and 21.2% of the threshold of toxicological concern for alternariol and alternariol monomethyl-ether, respectively. The results highlighted the extensive presence of zearalenone-derived forms and *Alternaria* toxins in ready-to-eat nut products. The relevance showed in this study suggests the inclusion of these mycotoxins in analytical methods to perform more realistic risk assessments, even more when only little toxicological data are available for setting a proper legislation.

## 4. Materials and Methods

### 4.1. Chemicals and Reagents

Methanol (MeOH), acetonitrile water, and formic acid (FA) for LC mobile phase (HPLC grade) were purchased from Merck (Darmstadt, Germany). Sodium chloride (NaCl), ammonium formate (NH_4_HCO_2_), magnesium sulfate (MgSO_4_), and octadecyl carbon chain-bonded silica (C18) were acquired from Sigma Aldrich (Milan, Italy). Eighteen mycotoxin standards (purity >98%) including aflatoxins (AFB1, AFB2, AFG1, and AFG2), zearalenone (ZEN), α-zearalanol (α-ZAL), α-zearalenol (α-ZEL), β-zearalanol (β-ZAL), neosolaniol (NEO), T-2 toxin, HT-2 toxin, enniatins (A, A1, B, and B1), alternariol monomethyl ether (AME), and alternariol (AOH) were obtained from Sigma-Aldrich (Milan, Italy).

Stock solutions of each mycotoxin were built by dissolving 1 mg of solid reference standard in 1 mL of methanol. An intermediate mixed solution containing all the mycotoxins at a concentration of 30 μg/mL was obtained after mixing individual stock solutions and diluting in MeOH:H_2_O (70:30 *v*/*v*) 0.1% formic acid. Working standard solutions at 1.6, 0.4, 0.08 μg/mL were used for spiking experiments (fortification levels at 20, 5, and 1 µg/kg). All solutions were stored in safe conditions at −20 °C in screw-capped glass vials.

### 4.2. Sampling

Fifty-four commercially available nut products were randomly purchased from supermarkets located in the Campania Region, Southern Italy. Products were classified as walnuts (*n* = 22), pistachios (*n* = 15), and almonds (*n* = 17) and sent to the laboratory in their original packages. Samples were kept in dark and cool conditions as recommended by the manufacturer, and analyses were carried out within five days after receiving them.

### 4.3. Sample Preparation

A procedure previously developed by Cunha, Sá, and Fernandes [14] was used as the starting point, with some modifications. Briefly, 10 g of homogenized sample was introduced into a 50 mL Falcon tube (Conical Polypropylene Centrifuge Tube; Thermo Fisher Scientific, Milan, Italy) and 5 mL of distilled water and 5 mL of acetonitrile containing 1% formic acid (*v*/*v*) were added. The sample was vortexed (ZX3; VEPL Scientific, Usmate, Italy) for 2 min. Then, 0.5 g of sodium chloride and 2.0 g of anhydrous sulfate sodium were added. The tube was manually shaken for 1 min and then centrifuged (X3R Heraeus Multifuge; Thermo Fisher Scientific, Kalkberg, Germany) for 10 min at 4907× *g* at room temperature. The supernatant (1.5 mL) was transferred to a 15 mL Falcon tube containing 50 mg of C18 sorbent, then vortexed for 1 min and centrifuged for 3 min at 4907× *g* at room temperature. Lastly, 0.4 mL were collected, filtered through a 0.22 μm filter and injected into the UHPLC-Q-Orbitrap HRMS instrument.

### 4.4. UHPLC-Q-Orbitrap HRMS Analysis

Detection and quantification analysis were performed using a Dionex UltiMate^®^ 3000 system consisting of a quaternary UHPLC pump working at 1250 bar (125 MPa), a degassing system, an autosampler device, and a thermostatically controlled column coupled with a Q-Exactive mass spectrometer (ThermoFisher Scientific, Waltham, MA, USA). Chromatographic separation of analytes was carried out with a thermostated Luna Omega Polar C18 column (50 × 2.1 mm, 1.6 μm; Phenomenex, Torrance, CA, USA) kept at 30 °C. Water (A) and methanol (B), both containing 0.1% formic acid and 5 mM ammonium acetate were used as mobiles phases. The gradient profile started with 0% B for 1 min, increased to 95% B over 1 min and kept for 0.5 min. Next, the gradient linearly decreased to 75% B over 2.5 min and decreased again until 60% in 1 min. Lastly, the gradient went back to 0% in 0.5 min and held for 1.5 min for column re-equilibration. Total run time was 8 min with an injection volume of 5 μL and a flow rate of 0.4 mL/min.

The mass spectrometer operated in positive and negative ion modes by setting 2 scan events: Full ion MS and all ion fragmentation (AIF). The following settings were used in full MS mode: Resolution power of 35,000 FWHM (defined for *m*/*z* 200), automatic gain control (AGC) target 1 × 10^6^, scan range 100–1000 *m*/*z*, injection time set to 200 ms, and scan rate set at 2 scans/s. The ion source parameters were: Capillary temperature 290 °C, S-lens RF level 50, spray voltage 4 kV (-kV en ESI- mode), sheath gas pressure (N_2_ > 95%) 35, auxiliary gas (N_2_ > 95%) 10, and auxiliary gas heater temperature 305 °C. The AIF mode used the following settings in both modes: Scan time = 0.10 s, maximum injection time = 200 ms; mass resolving power = 17,500 FWHM, ACG target = 1 × 10^5^, scan range = 100–1000 *m*/*z*, retention time window 30s, and isolation window 5.0 *m*/*z*. The collision energy and Orbitrap-MS parameters corresponding for each analyte were individually optimized in a previous work of our group [27]. The mass spectrometer was regularly calibrated using calibration solutions provided by ThermoFisher during three-day intervals and before each sequence. Retention time, elemental composition, theoretical and measured mass, accurate mass error, collision energy, and product ions for the analyzed compounds are shown in Table 6. A mass tolerance of 5 ppm was set for identification and confirmation of the molecular ion and both products. For accurate mass measurement, identification, and confirmation were performed at a mass tolerance of 5 ppm for the molecular ion and for both fragments. Xcalibur software, v.3.1.66.10. was used in order to analyze and process the data.

### 4.5. Validation Parameters

An in-house validation study was carried out for the three different matrices here analyzed following the EU Commission Decision 2002/657/EC guidelines referring to linearity, selectivity, trueness, intra-day precision (repeatability), inter-day precision (reproducibility), and sensitivity expressed as LOQs [28]. Linearity was determined by injecting a series of neat solvent and matrix-matched calibration curves at eight concentrations levels ranging from 0.2 to 200 ng/mL with a deviation of ≤20% for each calibration level. The coefficient of determination was calculated using the means of the least square approach. For evaluating a potential interference of the matrix, the slopes corresponding to each linear function were compared. The %SSE occurred when a deviation ≥20% was observed after comparing both slopes. The selectivity of the method was assessed by injecting ten blank samples, observing no peaks that could interfere in the same retention time area as the analytes, considering a mass error of 5 ppm. Trueness was evaluated through recovery studies, spiking three blank samples at three different fortification levels: 1, 5, and 20 µg/kg. The measurements were made during three non-consecutive days. Values ranging from 70 to 120% of recovery were considered as optimal. Precision was assessed in terms of repeatability (relative standard deviation after three determinations in a single day, RSD_r_) and reproducibility (relative standard deviation after determinations in triplicate on three non-consecutive days, RSD_R_). Sensitivity was determined through the LOQ for each analyte, which was established as the minimum concentration with a linear response that can be observed with a deviation ≤20% considering a mass error of 5 ppm.

### 4.6. Exposure Assessment

A deterministic approach was followed for performing the exposure assessment. The latest data consumption published by the Italian National Food Consumption Survey INRAN-SCAI 2005-06 were considered [29]. The commodities here analyzed were all included in the “nuts” category according to the survey, so exposure assessment was performed considering both the mean and the 95th percentile values. Population was divided in four age groups: Children (3–9.9 years) average consumption 6.4 g/day, P95 17.0 g/day; teenagers (10–17.9 years) average consumption 7 g/day, P95 22.3 g/day; adults (18–65 years) average consumption 9.5 g/day, P95 24.7 g/day; and elderly (65 < years) average consumption 11.1 g/day, P95 30.7 g/day. The mean body weights attached to each group were 26.1, 52.6, 69.7, and 70.1 kg, respectively, as detailed in the INRAN-SCAI 2005-06 survey. The probable daily intake (PDI) values were calculated using the next equation:

PDI = C × I/bw,(1)

where *C* represents the contamination (for the lower or upper bound) of each mycotoxin (µg/kg); *I* accounts for the mean or 95th percentile consumption established for each age group (g/day); and *bw* means the body weight assigned to its corresponding age group (kg). Once the PDIs were calculated, the tolerable daily intakes (TDIs) established for the detected mycotoxins were considered for performing the risk characterization. In case of finding a mycotoxin without any TDI assigned by the EFSA yet, a threshold of toxicological concern (TTC) was used. The risk characterization was calculated following the next equation:

%TDI = PDI/TDI × 100,(2)

Because of the high proportion of left-censored data, two scenarios of exposure were defined considering negative samples as zero or LOQ for the lower-bound and upper-bound, respectively.

### 4.7. Statistical Analysis

Validation experiments were conducted in triplicate and expressed as mean values alongside the corresponding relative standard deviation (RSD, %). The normality was evaluated through Saphiro–Wilk test and multivariant analysis was carried out using the Kruskal–Wallis test in order to compare the contamination levels among different matrices. A *p*-value < 0.05 was considered as significant. The statistical software package IBM SPSS version 25 was used for data analysis.

## Figures and Tables

**Table 1:** Method performance in almonds, walnuts, pistachios.

		Recovery (%) (RSD_R_ (%))			
Analyte	SSE (%)	20 ng/g	5 ng/g	1 ng/g	LOQ (ng/g)
almonds					
NEO	106	83 (12)	77 (16)	88 (7)	0.78
AFG2	101	88 (16)	82 (19)	78 (14)	0.20
AFG1	111	83 (20)	78 (20)	82 (13)	0.39
AFB2	106	93 (15)	94 (16)	83 (12)	0.20
AFB1	117	100 (10)	95 (15)	95 (5)	0.39
HT-2	115	105 (8)	89 (17)	79 (8)	0.78
A-ZAL	102	88 (11)	92 (12)	83 (10)	0.39
A-ZOL	109	92 (7)	88 (9)	87 (6)	0.78
AOH	105	81 (14)	73 (14)	71 (14)	0.20
T-2	114	104 (16)	87 (11)	89 (13)	0.78
B-ZAL	118	96 (10)	95 (13)	89 (10)	0.78
B-ZOL	98	95 (9)	90 (15)	76 (9)	0.20
ZON	120	94 (18)	81 (17)	86 (18)	0.20
AME	149	94 (15)	85 (16)	81 (15)	0.78
ENN B	77	94 (6)	90 (9)	98 (10)	0.78
ENN B1	106	106 (14)	84 (13)	74 (10)	0.78
ENN A1	111	87 (3)	86 (5)	95 (18)	0.39
ENN A	102	86 (12)	83 (13)	89 (12)	0.78
walnuts					
NEO	91	85 (9)	81 (14)	92 (10)	0.78
AFG2	93	85 (5)	78 (9)	82 (10)	0.20
AFG1	91	79 (5)	70 (8)	76 (11)	0.78
AFB2	103	91 (4)	84 (8)	79 (12)	0.39
AFB1	97	102 (6)	83 (12)	78 (10)	0.39
HT-2	107	97 (9)	88 (11)	76 (9)	0.78
A-ZAL	103	99 (10)	81 (12)	100 (9)	0.78
A-ZOL	110	88 (7)	78 (15)	75 (9)	0.78
AOH	105	83 (4)	71 (6)	74 (8)	0.20
T-2	96	86 (10)	84 (13)	79 (8)	0.78
B-ZAL	105	95 (5)	91 (11)	99 (12)	0.78
B-ZOL	97	94 (7)	85 (7)	95 (12)	0.20
ZON	100	81 (7)	78 (7)	73 (5)	0.20
AME	114	80 (7)	74 (9)	81 (15)	0.78
ENN B	74	101 (5)	90 (9)	84 (16)	0.78
ENN B1	99	88 (8)	89 (10)	76 (8)	0.78
ENN A1	96	105 (8)	99 (9)	76 (13)	0.78
ENN A	107	99 (8)	87 (15)	86 (8)	0.78
pistachios					
NEO	103	86 (10)	75 (14)	72 (8)	0.78
AFG2	82	106 (10)	82 (14)	82 (9)	0.39
AFG1	91	80 (11)	70 (11)	73 (6)	0.78
AFB2	87	113 (10)	103 (11)	97 (7)	0.39
AFB1	94	87 (18)	82 (18)	79 (13)	0.39
HT-2	105	91 (14)	85 (17)	88 (14)	0.78
A-ZAL	86	99 (13)	99 (16)	88 (17)	0.78
A-ZOL	97	83 (15)	85 (15)	89 (7)	0.78
AOH	83	84 (16)	77 (17)	83 (13)	0.39
T-2	85	92 (11)	98 (14)	85 (18)	0.78
B-ZAL	96	87 (10)	89 (15)	81 (9)	0.78
B-ZOL	112	92 (9)	92 (13)	89 (12)	0.78
ZON	118	104 (9)	105 (9)	107 (13)	0.20
AME	148	87 (10)	77 (16)	75 (14)	0.78
ENN B	115	99 (14)	92 (16)	83 (15)	0.78
ENN B1	116	102 (6)	94 (8)	96 (16)	0.78
ENN A1	118	105 (13)	99 (16)	96 (13)	0.78
ENN A	114	100 (14)	92 (17)	96 (15)	0.78

**Table 2:** Quantitative methods for multi-mycotoxin detection in several tree nut products.

Analytes (*n*)	Method	Sample Treatment	Sensitivity (µg/kg)	References
Aflatoxins (B1, B2, G1, G2), CIT, DON, FB1, FB2, FUS-X, HT-2, OTA, T-2, STE, ZEN (14)	UHPLC-MS/MS	QuEChERS-DLLME	0.61–150	Arroyo-Manzanares et al., 2013 [12]
Aflatoxins (B1, B2, G1, G2), BEA, DAS, enniatins (A, A1, B, B1), FB1, FB2, FB3, HT-2, OTA, T-2 (16)	LC-MS/MS	QuEChERS-SPE cartridge	0.2–45	Azaiez et al., 2014 [13]
Aflatoxins (B1, B2, G1, G2), DAS, 3AC-DON, 15AC-DON, DON, FB1, FB2, FUS-X, HT-2, NEO, OTA, T-2, ZEN (16)	LC-MS/MS	QuEChERS-Z-Sep^+^ + C18	1.25–5	Cunha et al., 2018 [14]
Aflatoxins (B1, B2, G1, G2), AME, AOH, BEA, enniatins (A, A1, B, B1), OTA, OTB, T-2, TEN, ZEN (16)	UPLC-MS/MS	QuEChERS-C18	0.1–5	Wang et al., 2018 [15]
3-ADON, aflatoxins (B1, B2, G1, G2, M1), DAS, ERGC1, ERGC2, FB1, FB2, GLI, HT-2, OTA, T-2, α-ZEL, ZEN (17)	Nano flow LC-HRMS	QuEChERS EMR-Lipid	0.05–5	Alcantara et al., 2019 [16]
Aflatoxins (B1, B2, G1, G2), α-ZEL, ZEN (6)	UHPLC-MS/MS	QuEChERS-C18	0.5–1	Hidalgo et al., 2019 [17]
Aflatoxins (B1, B2, G1, G2), AME, AOH, enniatins (A, A1, B, B2), HT-2, NEO, T-2, α-ZAL, α-ZEL, β-ZAL, β-ZEL, ZEN (18)	UHPLC-HRMS	QuEChERS-C18	0.2–0.78	Present work

AME: Alternariol monomethyl-ether; AOH: Alternariol; BEA: Beauvericin; CIT: Citrinin; DAS: Diacetoxyscirpenol; DLLME: Dispersive liquid–liquid microextraction; 3AC-DON: 3-acetyl-deoxynivalenol; 15AC-DON: 15-acetyl-deoxynivalenol; DON: Deoxynivalenol; EMR: Enhanced matrix removal; ERGC1: Ergocornine 1; ERGC2: Ergocornine 2; FB1: Fumonisin B1; FB2: Fumonisin B2; FB3: Fumonisin B3; FUS-X: Fusarenon-X; GLI: Gliotoxin; HRMS: High-resolution mass spectrometry; LC: Liquid chromatography; MS/MS: Tandem mass spectrometry; NEO: Neosolaniol; OTA: Ochratoxin A; OTB: Ochratoxin B; STE: Sterigmatocystin; TEN: Tentoxin; UHPLC: Ultra-high performance liquid chromatography; UPLC: Ultra performance liquid chromatography; α-ZAL: Alpha-zearalanol; α-ZEL: Alpha-zearalenol; β-ZAL: Beta-zearalanol; β-ZEL: Beta-zearalenol; ZEN: Zearalenone.

**Table 3:** Occurrence of mycotoxins in the analyzed tree nuts.

			Range (µg/kg)	
Analyte	Incidence (*n*, (%))	Mean (µg/kg)	Min	Max
*Almonds (n = 17)*				
AFB1	1 (6)	0.45	-	-
α-ZAL	3 (18)	3.99	3.70	4.54
α-ZEL	1 (6)	1.40	-	-
β-ZEL	2 (12)	0.54	0.46	0.62
AOH	2 (12)	0.35	0.34	0.37
*Walnuts (n = 22)*				
α-ZAL	2 (12)	2.18	2.13	2.24
β-ZAL	3 (18)	3.13	1.67	5.24
β-ZEL	4 (24)	0.39	0.3	0.55
ZEN	3 (18)	0.44	<LOQ	0.93
AOH	9 (53)	0.67	0.29	1.65
AME	3 (18)	1.63	1.13	1.95
ENN B1	1 (6)	1.30	-	-
*Pistachios (n = 15)*				
α-ZAL	2 (12)	25.75	2.16	49.35
β-ZAL	1 (6)	11.86	-	-
α-ZEL	2 (12)	1.50	1.26	1.74
β-ZEL	10 (59)	3.42	0.96	8.60
AOH	1 (6)	7.75	-	-

**Table 4:** Co-occurrence of mycotoxins in the analyzed tree nuts.

Combinations of Mycotoxins	Incidence (n, (%))
*Almonds (n = 17)*	
α-ZAL + α-ZEL	1 (6)
*Walnuts (n = 22)*	
AOH + α-ZAL	1 (6)
AOH + β-ZEL	1 (6)
AOH + ZEN	2 (12)
α-ZAL + AME	1 (6)
α-ZAL + β-ZAL	1 (6)
α-ZEL + β-ZEL	1 (6)
AOH + α-ZAL + ZEN	1 (6)
AOH + β-ZEL + AME	1 (6)
AOH + β-ZEL + β-ZAL + AME	1 (6)
*Pistachios (n = 15)*	
α-ZAL+ β-ZAL	1 (6)
β-ZAL + β-ZEL	1 (6)
α-ZEL + β-ZEL	1 (6)
AOH + β-ZEL	1 (6)

**Table 5:** Risk characterization of detected mycotoxins in different population groups based on the percentage of tolerable daily intake considering four different scenarios per group: Mean and 95th percentile consumption value combined with lower and upper bound of contamination.

				Risk Characterization (%TDI or %TTC)															
				Child				Teenager				Adult				Elderly			
		C (µg/kg)		Mean		P95th		Mean		P95th		Mean		P95th		Mean		P95th	
Mycotoxins	TDI or TTC (µg/kg bw/day)	LB	UB	LB	UB	LB	UB	LB	UB	LB	UB	LB	UB	LB	UB	LB	UB	LB	UB
∑ ZEN_d_	0.25	7.25	59.70	0.7	5.9	1.9	15.6	0.4	3.2	1.2	10.1	0.4	3.3	1.0	8.5	0.5	3.8	1.3	10.5
AOH	0.0025	0.10	0.48	0.8	4.8	2.8	12.4	0.4	2.4	1.6	8.0	0.4	2.8	1.6	6.8	0.8	3.2	1.6	8.4
AME	0.0025	0.25	0.82	2.4	8.0	6.4	21.2	1.2	4.4	4.4	14.0	1.2	4.4	3.6	11.6	1.6	5.2	4.4	14.4

∑ ZEN_d_: Sum of ZEN and its derived forms α-ZAL, α-ZEL, β-ZAL, β-ZEL; TDI: Tolerable daily intake; TTC: Threshold of toxicological concern; C: Contamination; LB: Lower bound; UP: Upper bound; P95th: 95th percentile.

**Table 6:** UHPLC-HRMS parameters corresponding to the here-analyzed mycotoxins.

Analyte	Retention Time (min)	Elemental Composition	Adduct Ion	Theoretical Mass (*m*/*z*)	Measured Mass (*m*/*z*)	Accuracy (Δ ppm)	Collision Energy (eV)	Product Ions (*m*/*z*)
NEO	4.25	C_19_H_26_O_8_	(M+NH_4_)^+^	400.19659	400.19632	−0.67	10	305.13803
								141.00530
AFG2	4.52	C_17_H_14_O_7_	(M+H)^+^	331.08123	331.08078	−1.36	37	313.07010
								245.08032
AFG1	4.55	C_17_H_12_O_7_	(M+H)^+^	329.06558	329.06549	−0.27	40	243.06467
								200.04640
AFB2	4.60	C_17_H_14_O_6_	(M+H)^+^	315.08631	315.08615	−0.51	36	287.09064
								259.05945
AFB1	4.64	C_17_H_12_O_6_	(M+H)^+^	313.07066	313.07053	−0.42	36	285.07489
								269.04373
HT−2	4.74	C_22_H_32_O_8_	(M+NH_4_)^+^	442.24354	442.24323	−0.70	27	263.12744
								215.10641
α-ZAL	4.81	C_18_H_26_O_5_	(M-H)^−^	321.17044	321.17065	0.65	29	259.09497
								91.00272
α-ZEL	4.83	C_18_H_24_O_5_	(M-H)^−^	319.15510	319.15500	−0.31	36	174.95604
								129.01947
T-2	4.84	C_24_H_34_O_9_	(M+NH_4_)^+^	484.25411	484.25430	0.39	23	215.10603
								185.09561
AOH	4.85	C_14_H_10_O_5_	(M-H)^−^	257.04555	257.04581	1.01	−32	215.03490
								213.05569
β-ZAL	4.94	C_18_H_26_O_5_	(M-H)^−^	321.17044	321.17059	0.47	40	259.09497
								91.00272
β-ZEL	4.97	C_18_H_24_O_5_	(M-H)^−^	319.15510	319.15500	−0.31	36	174.95604
								160.97665
ZEN	5.01	C_18_H_22_O_5_	(M+H)^+^	317.13945	317.13928	−0.54	−32	175.03989
								131.05008
AME	5.13	C_15_H_12_O_5_	(M-H)^−^	271.06120	271.06140	0.74	−36	256.03751
								228.04276
ENN B	5.56	C_33_H_57_N_3_O_9_	(M+NH_4_)^+^	657.44331	657.44348	0.26	50	214.14320
								196.13280
ENN B1	5.68	C_34_H_59_N_3_O_9_	(M+NH_4_)^+^	671.45986	671.45935	−0.76	48	214.14343
								196.13295
ENN A1	5.82	C_35_H_61_N_3_O_9_	(M+NH_4_)^+^	685.47461	685.47449	−0.18	48	228.15900
								210.14847
ENN A	5.99	C_36_H_63_N_3_O_9_	(M+NH_4_)^+^	699.49026	699.48987	−0.56	43	228.15900
								210.14847
